# A post-GWAS confirming the genetic effects and functional polymorphisms of *AGPAT3* gene on milk fatty acids in dairy cattle

**DOI:** 10.1186/s40104-020-00540-4

**Published:** 2021-02-01

**Authors:** Lijun Shi, Xin Wu, Yuze Yang, Zhu Ma, Xiaoqing Lv, Lin Liu, Yanhua Li, Feng Zhao, Bo Han, Dongxiao Sun

**Affiliations:** 1 Department of Animal Genetics, Breeding and Reproduction, College of Animal Science and Technology, Key Laboratory of Animal Genetics, Breeding and Reproduction of Ministry of Agriculture and Rural Affairs, National Engineering Laboratory for Animal Breeding, China Agricultural University, No. 2 Yuanmingyuan West Road, Haidian District, Beijing, 100193 China; 2 Institute of Animal Science, Chinese Academy of Agricultural Sciences, Beijing, 100193 China; 3 Beijing General Station of Animal Husbandry, Beijing, 100101 China; 4 Beijing Dairy Cattle Center, Beijing, 100192 China

**Keywords:** *AGPAT3*, Chinese Holstein, Genetic effects, Milk fatty acids, Potential causal mutation

## Abstract

**Background:**

People are paying more attention to the healthy and balanced diet with the improvement of their living standards. Milk fatty acids (FAs) have been reported that they were related to some atherosclerosis and coronary heart diseases in human. In our previous genome-wide association study (GWAS) on milk FAs in dairy cattle, 83 genome-wide significant single nucleotide polymorphisms (SNPs) were detected. Among them, two SNPs, ARS-BFGL-NGS-109493 and BTA-56389-no-rs associated with C18index (*P* = 0.0459), were located in the upstream of 1-acylglycerol-3-phosphate O-acyltransferase 3 (*AGPAT3*) gene. *AGPAT3* is involved in glycerol-lipid, glycerol-phospholipid metabolism and phospholipase D signaling pathways. Hence, it was inferred as a candidate gene for milk FAs. The aim of this study was to further confirm the genetic effects of the *AGPAT3* gene on milk FA traits in dairy cattle.

**Results:**

Through re-sequencing the complete coding region, and 3000 bp of 5′ and 3′ regulatory regions of the *AGPAT3* gene, a total of 17 SNPs were identified, including four in 5′ regulatory region, one in 5′ untranslated region (UTR), three in introns, one in 3′ UTR, and eight in 3′ regulatory region. By the linkage disequilibrium (LD) analysis with Haploview4.1 software, two haplotype blocks were observed that were formed by four and 12 identified SNPs, respectively. Using SAS9.2, we performed single locus-based and haplotype-based association analysis on 24 milk FAs in 1065 Chinese Holstein cows, and discovered that all the SNPs and the haplotype blocks were significantly associated with C6:0, C8:0 and C10:0 (*P* < 0.0001–0.0384). Further, with Genomatix, we predicted that four SNPs in 5′ regulatory region (g.146702957G > A, g.146704373A > G, g.146704618A > G and g.146704699G > A) changed the transcription factor binding sites (TFBSs) for transcription factors SMARCA3, REX1, VMYB, BRACH, NKX26, ZBED4, SP1, USF1, ARNT and FOXA1. Out of them, two SNPs were validated to impact transcriptional activity by performing luciferase assay that the alleles A of both SNPs, g.146704373A > G and g.146704618A > G, increased the transcriptional activities of *AGPAT3* promoter compared with alleles G (*P* = 0.0004).

**Conclusions:**

In conclusion, our findings first demonstrated the significant genetic associations of the *AGPAT3* gene with milk FAs in dairy cattle, and two potential causal mutations were detected.

**Supplementary Information:**

The online version contains supplementary material available at 10.1186/s40104-020-00540-4.

## Introduction

Milk fat is one of critical breeding objectives in dairy cattle. It is comprised of triglyceride (> 95%), diglyceride (2%), phospholipids (1%), cholesterol (0.05%) and small amount of free fatty acids (FAs) (~ 0.1%) [1]. The main components of triglyceride are glycerin and FAs, in which, the FAs act as precursors for the formation of other aroma components, such as esters and alcohols [2]. For the various milk fatty acid traits in Holstein cows, the estimated heritability values have been reported to be 0.14–0.33 for saturated fatty acids (SFAs) and 0.08–0.29 for unsaturated fatty acids (UFAs) [3–7].

Genome-wide association study (GWAS) is a commonly used strategy to identify potential genetic variants underlying important complex traits in human and domestic animals. So far, some candidate genes and QTL regions for milk production traits have been detected with GWA studies in dairy cattle, such as *DLGAP1*, *AP2B1*, *SCD*, BTA11 (1.59–3.37 Mb), and BTA3 (70.34–73.69 Mb) [8–13]. In our previous GWAS for milk FAs in Chinese Holstein cows, 83 genome-wide significant single nucleotide polymorphisms (SNPs) were detected in total [12], in which, two SNPs (ARS-BFGL-NGS-109493 and BTA-56389-no-rs) associated with C18index (*P* = 0.0459), were located in the upstream of 1-acylglycerol-3-phosphate O-acyltransferase 3 (*AGPAT3*) gene. In addition, we performed a joint GWAS for milk FAs in combined Chinese and Danish Holstein populations and found that a chromosome-wide significant QTL region of 146.29–146.31 Mb on BTA1 was associated with C18:0 [13]. The *AGPAT3* gene was nearby this region with approximately 400 kb. 1-acylglycerol-sn-glycero 3-phosphate acyltransferase (AGPAT), encoded by the *AGPAT3* gene*,* is one of the isoforms of AGPATs [14] and is involved in the glycerolipid (ko00561) and glycerophospholipid metabolisms (ko00564), and phospholipase D signaling pathway (ko04072). Mammalian AGPAT catalyzed the acylation of lysophosphatidic acid to form the phosphatidic acid that was the precursor of all glycerplipids [14]. Therefore, it was implied that the *AGPAT3* gene was a promising candidate gene for milk FA traits in dairy cattle. The purpose of the present study was to further detect whether the *AGPAT3* gene had significant genetic effects on milk FAs in a Chinese Holstein cow population.

## Materials and methods

### Animals and phenotypic data

In this study, a total of 1065 Chinese Holstein cows were used as descripted in a previous research [15], which milk samples were collected in Beijing Dairy Cattle Center (www.bdcc.com.cn) to measure milk FA contents. With the gas chromatography method, a total of 16 milk FAs (C6:0, C8:0, C10:0, C11:0, C12:0, C13:0, C14:0, C14:1, C15:0, C16:0, C16:1, C17:0, C17:1, C18:0, C18:1*cis*-9 and C20:0) were measured as the weight proportion of total fat weight [12]. With the phenotypes, we calculated five indices based on the formula$\frac{cis\hbox{-} 9\mathrm{unsaturated}}{cis\hbox{-} 9\mathrm{unsaturated}+\mathrm{saturated}}\times 100$ [16]: C14index = $\frac{\mathrm{C}14:1}{\mathrm{C}14:1+\mathrm{C}14:0}\times 100$, C16index = $\frac{\mathrm{C}16:1}{\mathrm{C}16:1+\mathrm{C}16:0}\times 100$, C17index = $\frac{\mathrm{C}17:1}{\mathrm{C}17:1+\mathrm{C}17:0}\times 100$, C18index = $\frac{cis\hbox{-} 9}{\mathrm{C}18:1 cis\hbox{-} 9+\mathrm{C}18:0}\times 100$, and total index = $\frac{\mathrm{C}14:1+\mathrm{C}16:1+\mathrm{C}17:1+\mathrm{C}18:1 cis\hbox{-} 9}{\mathrm{C}14:1+\mathrm{C}14:0+\mathrm{C}16:1+\mathrm{C}16:0+\mathrm{C}17:1+\mathrm{C}17:0+\mathrm{C}18:1 cis\hbox{-} 9+\mathrm{C}18:0}\times 100$. In addition, the summarized SFA and UFA, and SFA/UFA were obtained as well.

### SNP identification and genotyping

Based on the genomic sequence of bovine *AGPAT3* gene (Gene ID: 506607), 14 pairs of primers (Table S1) were designed by the Primer 3 version 4.0 (http://bioinfo.ut.ee/primer3-0.4.0/) and were synthesized in the Beijing Genomics Institute (Beijing, China) to amplify all the exons with partial adjacent intron region, and 3000 bp of 5′ and 3′ regulatory regions. As previously descripted [15], two DNA pools were constructed and the polymerase chain reaction (PCR) amplifications were performed with each DNA pool as template. To identify potential polymorphisms, the PCR amplification products were bi-directionally sequenced with an ABI3730XL DNA analyzer (Applied Biosystems, Foster, CA, USA). Then, the identified SNPs were genotyped for the 1065 cows by the matrix-assisted laser-desorption/ionization time of flight mass spectrometry (MALDI-TOF MS, Sequenom MassARRAY, Bioyong Technologies Inc., HK).

### Linkage disequilibrium (LD) and association analyses

We estimated the LD among the identified SNPs of *AGPAT3* gene with Haploview 4.1 (Broad Institute, Cambridge, MA, USA).

For association analysis, the 1065 cows were traced back to three-generation pedigrees to construct the kinship matrix (A-matrix) by SAS 9.2 (SAS institute, Cary, NC, USA), so that 3335 individuals were totally included. Single-locus and haplotype-based associations with 24 kind of milk FAs were performed by the following mixed animal model with SAS 9.2:

$$ {Y}_{ijklm}=\upmu +{G}_i+{h}_j+{l}_k+{a}_l+\mathrm{b}\times {M}_m+{e}_{ijklm} $$

Here, *Y*_*ijklm*_ is the phenotypic value of each milk fatty acid trait; *μ* is the overall mean; *G*_*i*_ is the fixed effect corresponding to the genotype or haplotype combination of individual *i*; *h*_*j*_ (*j *= 1–23) and *l*_*k*_ (*k* = 1–4) were the fixed effect of farm *j* and stage of lactation l*l*, respectively; *a*_*l*_ is the random polygenic effect; *M*_*m*_ (*m* = 1–293) is the fixed effect of age at calving *m*; *b* is the regression coefficient of covariate *M*; and *e*_*ijklm*_ is the random residual. Further, we calculated the additive effect (a), dominant effect (d), and allele substitution effect (α) according to $a=\frac{AA- BB}{2},d= AB-\frac{AA+ BB}{2}, and\alpha =a+d\left(q-p\right)$ [17]. Here, AA, AB and BB were the least square means of milk FAs corresponding to the genotypes, and *p* and *q* were the frequencies of allele A and B, respectively.

### Prediction of changes of transcription factor binding sites (TFBSs) caused by the SNPs in 5′ regulatory region

We used the Genomatix software suite v3.9 (http://www.genomatix.de/cgi-bin/welcome/welcome.pl?s=d1b5c9a9015b02bb3b1a806f9c03293f) [18] to predict whether the four SNPs in 5′ regulatory region of *AGPAT3* (g. 146702957G > A, g.146704373A > G, g.146704618A > G, and g.146704699G > A) changed the TFBSs.

### Recombinant plasmid construction and luciferase assay

**Fig. 1:**
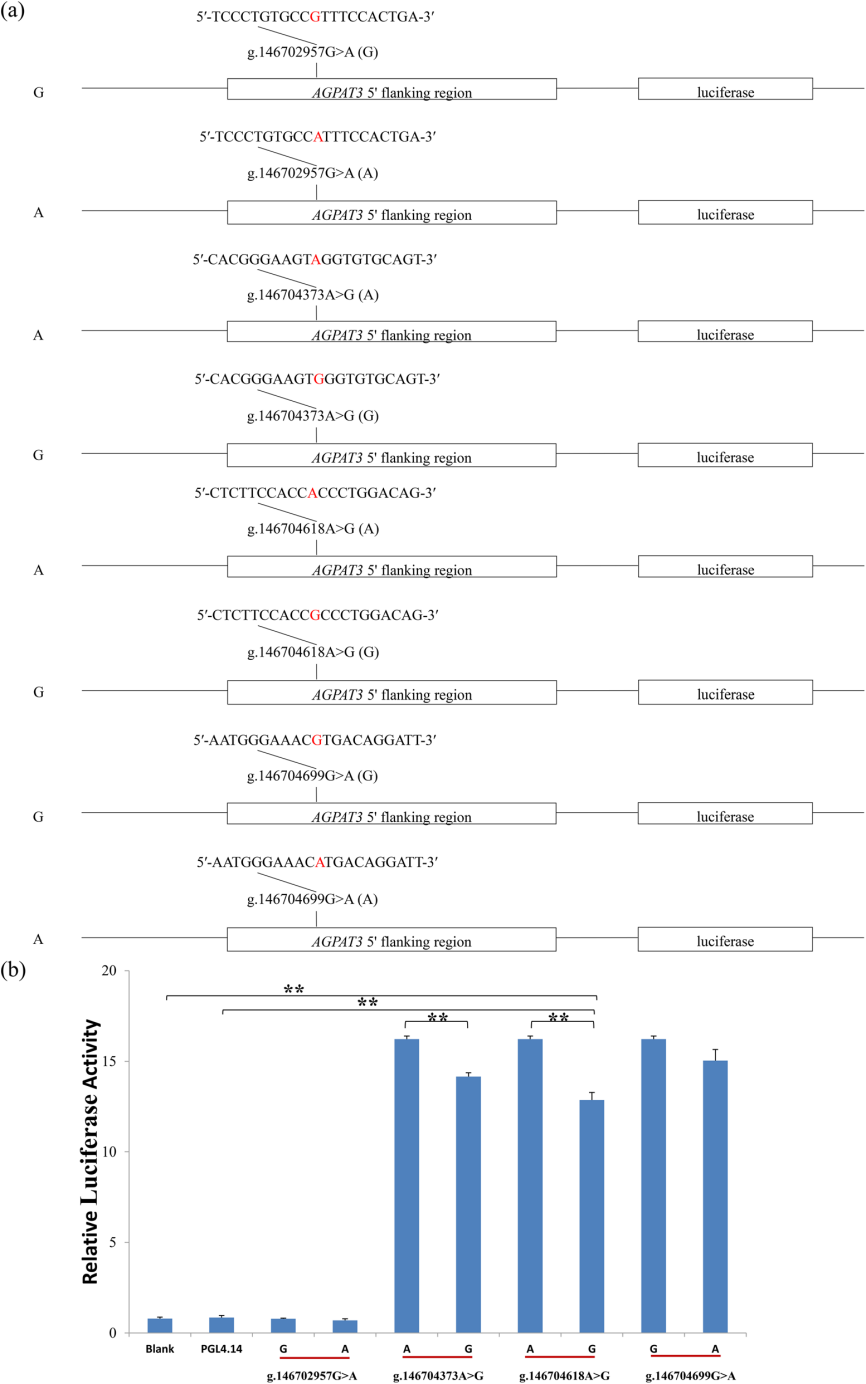
Luciferase assay. **a** Sketches of recombinant plasmids with g.146702957G > A, g.146704373A > G, g.146704618A > G, and g.146704699G > A in the 5′ flanking region of *AGPAT3*gene. The nucleotides in red highlight refer to the SNP. **b** Luciferase assay analysis of the recombinant plasmids in HEK293 cells. Blank: Blank cells. PGL4.14: Empty vector. ^**^: *P* < 0.01

To detect the allele-specific effects of the SNPs g.146702957G > A, g.146704373A > G, g.146704618A > G, and g.146704699G > A on the transcriptional activity of *AGPAT3* gene, eight luciferase reporter gene fragments (G and A of g. 146702957G > A; A and G of g.146704373A > G; A and G of g.146704618A > G; and G and A of g.146704699G > A) corresponding to the eight alleles of the four SNPs (Fig. 1a) were designed and synthesized (Genewiz, Suzhou, China). The eight fragments with the KpnI and Nhel restriction sites at the 5′ and 3′ termini, respectively, were cloned into the pGL4.14 luciferase assay vector (Promega, Madison, USA). In addition, all the plasmids were purified by the Endo-free Plasmid DNA Mini Kit II (OMEGA, omega bio-tek, Norcross, Georgia, USA), and were sequenced to confirm the integrity of each construct’s insertion.

The human embryonic kidney (HEK) 293 T cells were cultured with Dulbecco′s modified Eagle′s medium (Gibco, Life Technologies) and 10% fetal bovine serum (Gibco) at 37 °C in a humidified incubator containing 5% CO_2_. Before transfection, about 2 × 10^5^ cells were seeded in each 24-well plate. For eight luciferase reporter gene fragments of g. 146702957G > A, g.146704373A > G, g.146704618A > G and g.146704699G > A, 500 ng constructed plasmid was co-transfected along with 10 ng pRL-TK Renilla luciferase reporter vector (Promega) into each well. All the experiments were performed in three replicates for each construct. Approximate 48 h after transfection, the cells were harvested and the activity of both firefly and Renilla luciferases were measured with a Dual-Luciferase Reporter Assay System (Promega) on a Modulus microplate multimode reader (Turner Biosystems, CA, USA). The average statistic of three replicates were calculated as the normalized luciferase data (Firefly/Renilla).

## Results

### Identification of SNPs

A total of 17 SNPs of the *AGPAT3* gene was detected in this study (Table 1), which consisted of four (g.146702957G > A, g.146704373A > G, g.146704618A > G and g.146704699G > A) in 5′ flanking region, one (g.146705692G > A) in 5′ untranslated region (UTR), three (g.146725085 T > C, g.146726096A > G and g.146729107A > C) in introns, one (g.146735090G > T) in 3′ UTR, and eight (g.146737188C > T, g.146737545G > A, g.146737748 T > C, g.146737849C > T, g.146737879 T > G, g.146737916 T > C, g.146737946C > T and g.146738055G > A) in 3′ flanking region. The genotype and allele frequencies of the identified SNPs were presented in Table 1.

**Table 1:** Information of 17 SNPs of *AGPAT3* gene with genotypic and allelic frequencies

SNP name	Location	Position (UMD 3.1.1)	GenBank No.	Origin	Genotypes	NO.	Frequency	Allele	Frequency
g. 146702957G > A	5′ flanking region	Chr1: 146702957	rs210638665	NCBI	AA	78	0.0741	A	0.2692
					GG	564	0.5356	G	0.7308
					GA	411	0.3903		
g. 146704373A > G	5′ flanking region	Chr1: 146704373	rs209442459	NCBI	AA	560	0.5369	A	0.7311
					GG	78	0.0748	G	0.2689
					GA	405	0.3883		
g. 146704618A > G	5′ flanking region	Chr1: 146704618	rs110551271	NCBI	AA	520	0.4910	A	0.7030
					GG	90	0.0850	G	0.2970
					GA	449	0.4240		
g. 146704699G > A	5′ flanking region	Chr1: 146704699	rs110278717	NCBI	AA	113	0.1076	A	0.3219
					GG	487	0.4638	G	0.6781
					GA	450	0.4286		
g. 146705692G > A	5′ UTR	Chr1: 146705692	rs43281404	NCBI	AA	10	0.0095	A	0.1360
					GG	778	0.7374	G	0.8640
					AG	267	0.2531		
g. 146725085 T > C	Intron-5	Chr1: 146725085	rs110897007	NCBI	GG	634	0.6021	G	0.7835
					TT	37	0.0351	T	0.2165
					GT	382	0.3628		
g. 146726096A > G	Intron-6	Chr1: 146726096	rs378285374	NCBI	CC	380	0.3647	C	0.6027
					TT	166	0.1593	T	0.3973
					CT	496	0.4760		
g. 146729107A > C	Intron-7	Chr1: 146729107	rs43276015	NCBI	AA	166	0.1582	A	0.3990
					GG	378	0.3603	G	0.6010
					GA	505	0.4814		
g. 146735090G > T	3′ UTR	Chr1:146735090	rs379405887	NCBI	CC	169	0.1608	C	0.4015
					TT	376	0.3578	T	0.5985
					CT	506	0.4814		
g. 146737188C > T	3′ flanking region	Chr1: 146737188	rs383583298	NCBI	CC	376	0.3588	C	0.6007
					TT	165	0.1574	T	0.3993
					CT	507	0.4838		
g. 146737545G > A	3′ flanking region	Chr1: 146737545	rs43766238	NCBI	GG	166	0.1587	G	0.3991
					TT	377	0.3604	T	0.6009
					GT	503	0.4809		
g. 146737748 T > C	3′ flanking region	Chr1: 146737748	rs43760756	NCBI	CC	169	0.1608	C	0.4015
					TT	376	0.3578	T	0.5985
					TC	506	0.4814		
g. 146737849C > T	3′ flanking region	Chr1: 146737849	rs43760757	NCBI	CC	371	0.3540	C	0.5973
					TT	167	0.1594	T	0.4027
					CT	510	0.4866		
g. 146737879 T > G	3′ flanking region	Chr1: 146737879	rs43760758	NCBI	AA	12	0.0114	A	0.1360
					GG	780	0.7393	G	0.8640
					AG	263	0.2493		
g. 146737916 T > C	3′ flanking region	Chr1: 146737916	rs43760759	NCBI	CC	536	0.5174	C	0.7210
					TT	78	0.0753	T	0.2790
					TC	422	0.4073		
g. 146737946C > T	3′ flanking region	Chr1: 146737946	rs43760760	NCBI	AA	894	0.8442	A	0.9193
					GG	6	0.0057	G	0.0807
					AG	159	0.1501		
g. 146738055G > A	3′ flanking region	Chr1: 146738055	rs382304348	NCBI	AA	208	0.2006	A	0.4619
					CC	287	0.2768	C	0.5380
					CA	542	0.5227		

Note: *NO*. Number of cows with corresponding genotypes. *UTR* Untranslated region

### Estimation of LD among the identified SNPs of *AGPAT3*

**Fig. 2:**
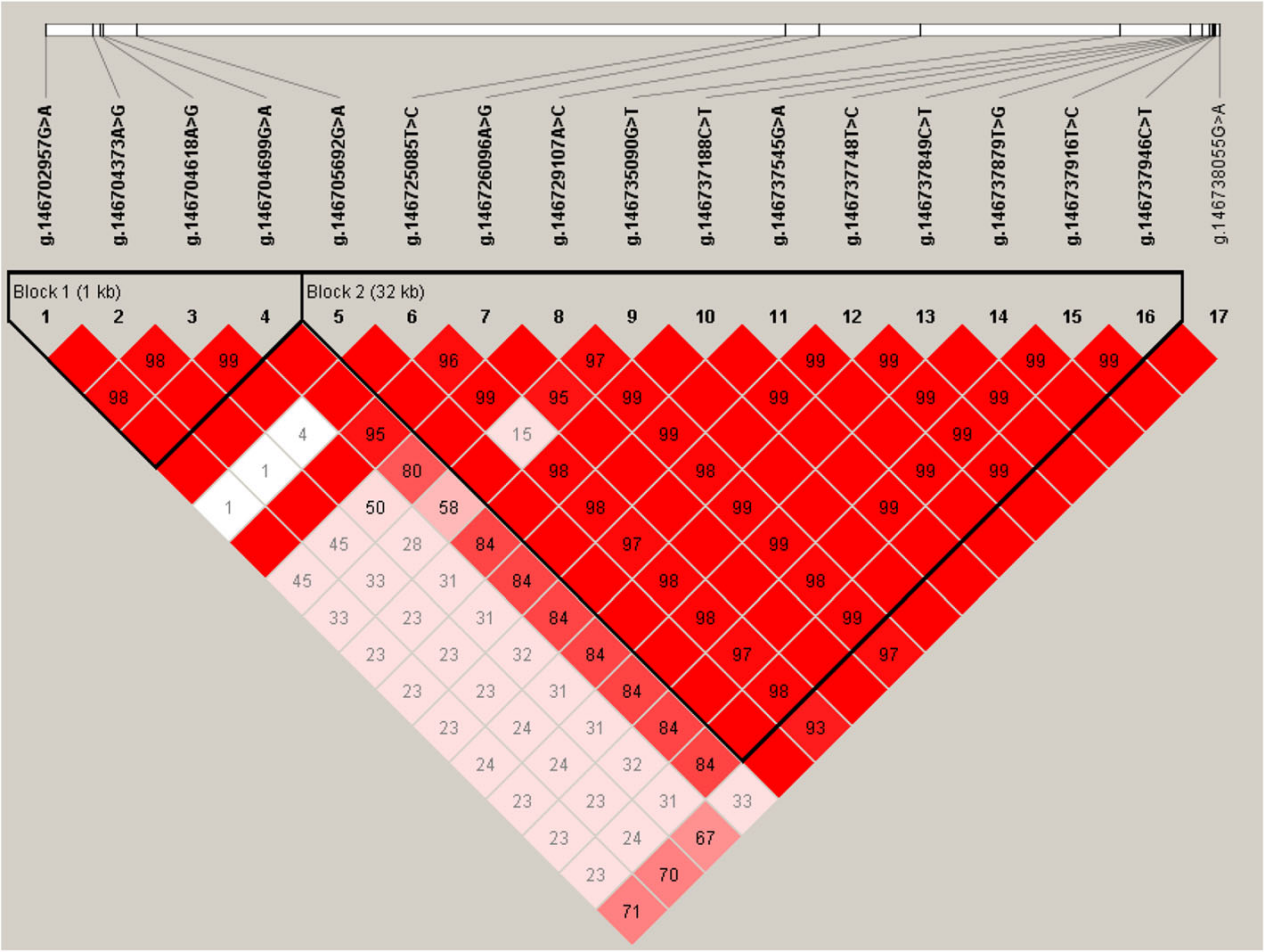
Linkage disequilibrium (LD) among the 17 SNPs of *AGPAT3* gene. The blocks indicate haplotype blocks, and the text above the horizontal number is the SNP names. The values in boxes are pairwise SNP correlations (D´), while the bright red boxes without numbers inffer complete LD (D´ = 1). The boxes have the greater LD with the brigther red

We used the haploview 4.1 to estimate the LD among these 17 SNPs, and observed two haplotype blocks (Fig. 2) that was formed by four and 12 SNPs, respectively. The haplotype block 1 included four haplotype combinations, namely, H1: GAAG (38%), H2: GAAA (32.2%), H3: AGGG (26.6%), and H4: GAGG (3%), and the haplotype block 2 had six haplotype combinations: H1 = GTAAGCGTCTTC, H2 = GCACGTACTGCT, H3 = GCAATCGTCTTC, H4 = ACACGCGTCTTC, H5 = GTGATCGTCTTC, and H6 = GCAAGCGTCTTC with their frequencies of 20%, 39.8%, 13.4%, 13.4%, 7.9% and 4.1%.

### Associations between *AGPAT3* and milk FAs

The associations of the 17 SNPs with 24 milk FAs were summarized in Table 2. Among these SNPs, 17 were strongly associated with C6:0 (*P* < 0.0001–0.0004) and C8:0 (*P* < 0.0001–0.0384); 14 were significantly associated with total index (*P* < 0.0001–0.0318); ten were significantly associated with C10:0 (*P* = 0.0016–0.0151); nine were strongly associated with C17:1 (*P* < 0.0001–0.0149); seven were significantly associated with C20:0 (*P* < 0.0001–0.0072); five had significant associations with C14:0 (*P* < 0.0001–0.0477); five were strongly associated with C17index (*P* = 0.0006–0.0389); five had strong associations with C18:1*cis*-9 (*P* < 0.0001–0.0258); three had significant associations with C18:0 (*P* = 0.0020–0.0246); three had strong associations with SFA (*P* < 0.0001–0.0434); two were significantly associated with C17:0 (*P* = 0.0212–0.0413); two were significantly associated with UFA (*P* < 0.0001 and *P* = 0.0386); one was strongly associated with C18index (*P* = 0.0249); and one had significant association with SFA/UFA (*P* = 0.0005). However, no significant association was found with C11:0, C12:0, C13:0, C14:1, C15:0, C16:0, C16:1, C14index and C16index (*P* > 0.05).

**Table 2:** Association between 17 SNPs and milk fatty acid traits in Chinese Holstein cows (LSM ± SE)

SNP	Genotype (No.)	C6:0, %	C8:0, %	C10:0, %	C11:0, %	C12:0, %	C13:0, %	C14:0, %	C14:1, %	C15:0, %	C16:0, %	C16:1, %	C17:0, %
g. 146702957G > A	AA(66–73)	0.5840 ± 0.0204^A^	0.9946 ± 0.0175^A^	2.8618 ± 0.0492	0.0583 ± 0.0048	3.0058 ± 0.0652	0.0977 ± 0.0055	10.1091 ± 0.1114^a^	0.6665 ± 0.0323	0.9937 ± 0.0219	34.7663 ± 0.3048	1.3640 ± 0.0417	0.5631 ± 0.0055
	GG(473–509)	0.4264 ± 0.0119^B^	0.9187 ± 0.0106^B^	2.8278 ± 0.0319	0.0584 ± 0.0034	3.0015 ± 0.0410	0.1003 ± 0.0029	10.2460 ± 0.0695	0.6475 ± 0.0178	0.9909 ± 0.0120	34.8480 ± 0.1750	1.3327 ± 0.0247	0.5693 ± 0.0031
	GA(337–367)	0.5226 ± 0.0125^C^	0.9914 ± 0.0111^A^	2.8646 ± 0.0330	0.0586 ± 0.0035	3.0068 ± 0.0425	0.0985 ± 0.0030	10.3670 ± 0.0713^b^	0.6730 ± 0.0189	0.9968 ± 0.0128	34.8295 ± 0.1822	1.3238 ± 0.0255	0.5653 ± 0.0032
	*P*	<.0001^**^	<.0001^**^	0.2626	0.9962	0.9854	0.7895	0.0095^**^	0.2843	0.8721	0.9581	0.5783	0.2642
g. 146704373A > G	AA(470–505)	0.4101 ± 0.0120^A^	0.8829 ± 0.0106^A^	2.8096 ± 0.0318	0.0579 ± 0.0035	3.0240 ± 0.0410	0.0997 ± 0.0028	10.2422 ± 0.0699	0.6411 ± 0.0179	0.9906 ± 0.0121	34.8165 ± 0.1762	1.3326 ± 0.0246	0.5696 ± 0.0031
	GG(66–73)	0.5641 ± 0.0205^B^	0.9368 ± 0.0173^B^	2.8117 ± 0.0499	0.0571 ± 0.0047	3.0067 ± 0.0652	0.0967 ± 0.0055	10.1221 ± 0.1115	0.6567 ± 0.0324	0.9887 ± 0.0222	34.8297 ± 0.2999	1.3626 ± 0.0423	0.5643 ± 0.0055
	GA(332–361)	0.5002 ± 0.0127^C^	0.9533 ± 0.0111	2.8525 ± 0.0334	0.0574 ± 0.0036	3.0271 ± 0.0429	0.0975 ± 0.0030	10.3515 ± 0.0717	0.6651 ± 0.0190	0.9959 ± 0.0129	34.8408 ± 0.1846	1.3192 ± 0.0260	0.5641 ± 0.0033
	*P*	<.0001^**^	<.0001^**^	0.1676	0.9561	0.9398	0.7032	0.0261^*^	0.3318	0.8781	0.9871	0.5151	0.1370
g. 146704618A > G	AA(437–474)	0.4167 ± 0.0121^A^	0.9070 ± 0.0107^A^	2.8322 ± 0.0323^A^	0.0579 ± 0.0035	3.0223 ± 0.0414	0.0998 ± 0.0029	10.2405 ± 0.0701	0.6421 ± 0.0181	0.9925 ± 0.0122	34.7496 ± 0.1778	1.3183 ± 0.0250	0.5698 ± 0.0031^a^
	GG(76–84)	0.5594 ± 0.0193^Ba^	0.9726 ± 0.0164^B^	2.8859 ± 0.0473	0.0582 ± 0.0045	3.0483 ± 0.0612	0.0982 ± 0.0052	10.2407 ± 0.1056	0.6692 ± 0.0300	0.9926 ± 0.0207	34.6202 ± 0.2865	1.3507 ± 0.0398	0.5638 ± 0.0052
	GA(368–396)	0.5155 ± 0.0123^Bb^	1.0007 ± 0.0109^B^	2.9155 ± 0.0324^B^	0.0588 ± 0.0035	3.0496 ± 0.0421	0.0997 ± 0.0030	10.3059 ± 0.0708	0.6706 ± 0.0185	0.9956 ± 0.0125	34.8113 ± 0.1797	1.3120 ± 0.0251	0.5621 ± 0.0032^b^
	*P*	<.0001^**^	<.0001^**^	0.0016^**^	0.8984	0.6596	0.9495	0.4364	0.1903	0.9604	0.7525	0.5678	0.0212^*^
g. 146704699G > A	AA(97–102)	0.4391 ± 0.0173^A^	0.9396 ± 0.0151^A^	2.8484 ± 0.0435	0.0590 ± 0.0043	3.0193 ± 0.0569	0.0979 ± 0.0047	10.3492 ± 0.0976	0.6572 ± 0.0273	0.9839 ± 0.0189	34.6861 ± 0.2593	1.2832 ± 0.0362	0.5629 ± 0.0048
	AG(366–402)	0.4614 ± 0.0125^A^	0.9274 ± 0.0108^A^	2.8053 ± 0.0325^A^	0.0574 ± 0.0035	3.0216 ± 0.0421	0.0972 ± 0.0030	10.2789 ± 0.0714	0.6434 ± 0.0185	0.9898 ± 0.0125	34.8492 ± 0.1810	1.3158 ± 0.0253	0.5682 ± 0.0032
	GG(412–443)	0.5119 ± 0.0124^B^	0.9886 ± 0.0110^B^	2.8843 ± 0.0330^B^	0.0591 ± 0.0035	3.0363 ± 0.0417	0.1006 ± 0.0030	10.3046 ± 0.0707	0.6607 ± 0.0185	1.0021 ± 0.0126	34.7333 ± 0.1806	1.3390 ± 0.0252	0.5647 ± 0.0032
	*P*	<.0001^**^	<.0001^**^	0.0040^**^	0.6878	0.8808	0.5248	0.6869	0.5672	0.4571	0.6735	0.2175	0.3310
g. 146705692G > A	AA(9)	0.2969 ± 0.0462^A^	0.8508 ± 0.0393	2.7974 ± 0.1115	0.0544 ± 0.0099	3.0099 ± 0.1491	0.1107 ± 0.0136	9.8944 ± 0.2514	0.6254 ± 0.0768	0.9965 ± 0.0536	35.1757 ± 0.7126	1.2628 ± 0.0991	0.5820 ± 0.0133
	GG(638–690)	0.4844 ± 0.0114^B^	0.9413 ± 0.0103	2.7966 ± 0.0307	0.0577 ± 0.0034	3.0048 ± 0.0393	0.0975 ± 0.0026	10.1890 ± 0.0666^A^	0.6495 ± 0.0168	0.9888 ± 0.0113	34.8014 ± 0.1658	1.3316 ± 0.0233	0.5665 ± 0.0029
	GA(230–250)	0.4644 ± 0.0140^B^	0.9309 ± 0.0120	2.8012 ± 0.0361	0.0596 ± 0.0037	3.0349 ± 0.0459	0.1021 ± 0.0035	10.4710 ± 0.0777^B^	0.6425 ± 0.0211	1.0037 ± 0.0144	34.8758 ± 0.2043	1.3301 ± 0.0284	0.5663 ± 0.0036
	*P*	<.0001^**^	0.0384^*^	0.9829	0.6338	0.6684	0.2420	<.0001^**^	0.8836	0.4783	0.7941	0.7785	0.4920
g. 146725085 T > C	CC(449–486)	0.4525 ± 0.0121^A^	0.9110 ± 0.0108^A^	2.8094 ± 0.0324	0.0587 ± 0.0035	2.9767 ± 0.0417	0.0991 ± 0.0029	10.2790 ± 0.0705	0.6482 ± 0.0180	0.9924 ± 0.0123	34.9341 ± 0.1775	1.3135 ± 0.0250	0.5675 ± 0.0031
	CT(347–376)	0.5018 ± 0.0126^B^	0.9664 ± 0.0111^B^	2.8207 ± 0.0336	0.0576 ± 0.0036	2.9839 ± 0.0430	0.0981 ± 0.0030	10.1990 ± 0.0720	0.6559 ± 0.0189	0.9954 ± 0.0129	34.7645 ± 0.1858	1.3526 ± 0.0260	0.5690 ± 0.0033
	TT(65–70)	0.4274 ± 0.0202^A^	0.9116 ± 0.0170^A^	2.8365 ± 0.0487	0.0610 ± 0.0047	3.0082 ± 0.0641	0.1018 ± 0.0054	10.2449 ± 0.1100	0.6607 ± 0.0313	1.0009 ± 0.0216	34.7673 ± 0.2953	1.2804 ± 0.0416	0.5651 ± 0.0054
	*P*	<.0001^**^	<.0001^**^	0.7754	0.6288	0.8546	0.7757	0.3190	0.8512	0.9063	0.5001	0.0673	0.7081
g. 146726096A > G	AA(740–804)	0.4677 ± 0.0113^A^	0.9597 ± 0.0101^A^	2.8758 ± 0.0304	0.0578 ± 0.0034	3.0394 ± 0.0390	0.0996 ± 0.0026	10.3084 ± 0.0657^a^	0.6553 ± 0.0166	0.9947 ± 0.0111	34.9294 ± 0.1634	1.3251 ± 0.0230	0.5646 ± 0.0028
	AG(136–144)	0.4266 ± 0.0157^Bb^	0.9216 ± 0.0136^B^	2.8526 ± 0.0395	0.0557 ± 0.0040	3.0084 ± 0.0521	0.0974 ± 0.0041	10.1355 ± 0.0879^b^	0.6513 ± 0.0242	0.9798 ± 0.0166	34.4974 ± 0.2316	1.3088 ± 0.0324	0.5732 ± 0.0042
	GG(4–5)	0.6162 ± 0.0686^a^	0.9142 ± 0.0513	3.0515 ± 0.1473	0.0701 ± 0.0129	3.1630 ± 0.1962	0.0981 ± 0.0180	10.5609 ± 0.3391	0.5784 ± 0.1024	0.9762 ± 0.0715	34.9285 ± 0.9476	1.2342 ± 0.1318	0.5561 ± 0.0178
	*P*	0.0004^**^	0.0015^**^	0.3415	0.4269	0.5976	0.8364	0.0314^*^	0.7439	0.5894	0.0865	0.6686	0.0541
g. 146729107A > C	AA(166–183)	0.5256 ± 0.0149^Aa^	0.9758 ± 0.0127^A^	2.8547 ± 0.0375^b^	0.0565 ± 0.0039	2.9831 ± 0.0487	0.0988 ± 0.0037	10.1650 ± 0.0823	0.6707 ± 0.0228	0.9933 ± 0.0156	34.7045 ± 0.2154	1.3271 ± 0.0304	0.5650 ± 0.0039
	CC(240–260)	0.4039 ± 0.0138^B^	0.8846 ± 0.0119^B^	2.7656 ± 0.0356^Aa^	0.0564 ± 0.0037	2.9740 ± 0.0458	0.0988 ± 0.0034	10.2943 ± 0.0782	0.6559 ± 0.0207	0.9904 ± 0.0142	34.7739 ± 0.2011	1.3319 ± 0.0282	0.5718 ± 0.0036^a^
	CA(461–492)	0.4901 ± 0.0121^Ab^	0.9650 ± 0.0108^A^	2.8525 ± 0.0323^B^	0.0587 ± 0.0035	3.0187 ± 0.0413	0.0984 ± 0.0029	10.2937 ± 0.0696	0.6552 ± 0.0180	0.9991 ± 0.0121	34.8408 ± 0.1767	1.3357 ± 0.0247	0.5640 ± 0.0031^b^
	*P*	<.0001^**^	<.0001^**^	0.0020^**^	0.5029	0.3680	0.9905	0.1253	0.7282	0.7714	0.7516	0.9431	0.0413^*^
g. 146735090G > T	GG(524–568)	0.4596 ± 0.0118^A^	0.9428 ± 0.0105	2.8328 ± 0.0315	0.0589 ± 0.0034	3.0735 ± 0.0404	0.1001 ± 0.0027	10.3081 ± 0.0686	0.6514 ± 0.0176	0.9965 ± 0.0118	34.8202 ± 0.1713	1.3414 ± 0.0240	0.5697 ± 0.0030
	GT(326–349)	0.4480 ± 0.0127^A^	0.9288 ± 0.0113^A^	2.8230 ± 0.0334	0.0576 ± 0.0036	3.0090 ± 0.0430	0.0974 ± 0.0031	10.1970 ± 0.0723	0.6592 ± 0.0190	0.9898 ± 0.0129	34.7381 ± 0.1858	1.3009 ± 0.0260	0.5673 ± 0.0033
	TT(26–31)	0.7225 ± 0.0295^B^	0.9944 ± 0.0233^B^	2.9134 ± 0.0675	0.0537 ± 0.0061	3.0448 ± 0.0882	0.0929 ± 0.0078	10.0790 ± 0.1526	0.6209 ± 0.0446	0.9771 ± 0.0317	34.5408 ± 0.4264	1.3066 ± 0.0590	0.5726 ± 0.0078
	*P*	<.0001^**^	0.0069^**^	0.3563	0.5402	0.1107	0.4694	0.0477^*^	0.6492	0.7195	0.7122	0.1367	0.5951
g. 146737188C > T	CC(317–344)	0.5244 ± 0.0129^A^	0.9949 ± 0.0113^A^	2.8918 ± 0.0339^Aa^	0.0589 ± 0.0036	3.0228 ± 0.0438	0.0999 ± 0.0031	10.1935 ± 0.0741	0.6581 ± 0.0194	0.9988 ± 0.0132	34.7063 ± 0.1890	1.3351 ± 0.0264	0.5678 ± 0.0034
	CT(413–448)	0.4912 ± 0.0123^B^	0.9489 ± 0.0106^B^	2.8306 ± 0.0323^b^	0.0585 ± 0.0035	3.0423 ± 0.0413	0.0981 ± 0.0029	10.2310 ± 0.0697	0.6511 ± 0.0179	0.9955 ± 0.0122	34.8170 ± 0.1770	1.3330 ± 0.0248	0.5697 ± 0.0031
	TT(137–148)	0.4013 ± 0.0157^C^	0.9270 ± 0.0135^B^	2.7818 ± 0.0397^B^	0.0579 ± 0.0040	2.9847 ± 0.0514	0.0968 ± 0.0040	10.0919 ± 0.0876	0.6586 ± 0.0242	0.9790 ± 0.0166	34.6458 ± 0.2300	1.3487 ± 0.0321	0.5753 ± 0.0042
	*P*	<.0001^**^	<.0001^**^	0.0020^**^	0.9407	0.3982	0.7234	0.1655	0.8950	0.4650	0.6289	0.8567	0.1793
g. 146737545G > A	AA(137–148)	0.4117 ± 0.0157^A^	0.9200 ± 0.0135^Aa^	2.8094 ± 0.0396^a^	0.0565 ± 0.0040	2.9638 ± 0.0514	0.0975 ± 0.0040	10.1601 ± 0.0876	0.6582 ± 0.0241	0.9801 ± 0.0166	34.5818 ± 0.2305	1.3322 ± 0.0322	0.5706 ± 0.0042
	GG(316–343)	0.5261 ± 0.0130^B^	0.9867 ± 0.0113^B^	2.9008 ± 0.0339^b^	0.0584 ± 0.0036	3.0218 ± 0.0435	0.1007 ± 0.0031	10.2857 ± 0.0729	0.6558 ± 0.0194	1.0007 ± 0.0132	34.6776 ± 0.1877	1.3318 ± 0.0263	0.5654 ± 0.0033
	GA(419–455)	0.5097 ± 0.0121^B^	0.9491 ± 0.0107^Ab^	2.8496 ± 0.0319	0.0576 ± 0.0035	3.0188 ± 0.0412	0.0982 ± 0.0029	10.3073 ± 0.0697	0.6492 ± 0.0179	0.9951 ± 0.0122	34.8114 ± 0.1754	1.3239 ± 0.0247	0.5665 ± 0.0031
	*P*	<.0001^**^	<.0001^**^	0.0134^*^	0.7932	0.3853	0.6416	0.1234	0.8841	0.4545	0.4569	0.9184	0.4321
g. 146737748 T > C	CC(139–151)	0.3955 ± 0.0156^A^	0.9086 ± 0.0134^Aa^	2.8149 ± 0.0393^A^	0.0575 ± 0.0040	2.9404 ± 0.0514	0.0977 ± 0.0040	10.1822 ± 0.0867	0.6542 ± 0.0240	0.9773 ± 0.0165	34.6990 ± 0.2295	1.3348 ± 0.0317	0.5735 ± 0.0041
	CT(420–455)	0.4892 ± 0.0121^Ba^	0.9386 ± 0.0107^Ab^	2.8760 ± 0.0323	0.0583 ± 0.0035	3.0032 ± 0.0410	0.0994 ± 0.0028	10.3018 ± 0.0699	0.6476 ± 0.0179	0.9927 ± 0.0122	34.8666 ± 0.1762	1.3342 ± 0.0246	0.5674 ± 0.0031
	TT(314–341)	0.5161 ± 0.0130^Bb^	0.9793 ± 0.0113^B^	2.9154 ± 0.0336^B^	0.0585 ± 0.0036	2.9957 ± 0.0439	0.1015 ± 0.0031	10.2819 ± 0.0737	0.6574 ± 0.0194	0.9975 ± 0.0131	34.7461 ± 0.1888	1.3359 ± 0.0267	0.5663 ± 0.0034
	*P*	<.0001^**^	<.0001^**^	0.0106^*^	0.9369	0.3252	0.6124	0.2573	0.8346	0.4587	0.6140	0.9969	0.1867
g. 146737849C > T	CC(313–340)	0.5257 ± 0.0130^Aa^	0.9965 ± 0.0114^A^	2.8975 ± 0.0338^Aa^	0.0593 ± 0.0036	2.9970 ± 0.0438	0.1005 ± 0.0031	10.2313 ± 0.0740	0.6588 ± 0.0194	0.9977 ± 0.0133	34.6572 ± 0.1886	1.3386 ± 0.0265	0.5673 ± 0.0034
	CT(421–457)	0.4959 ± 0.0121^Ab^	0.9498 ± 0.0106^B^	2.8322 ± 0.0324^b^	0.0586 ± 0.0035	3.0009 ± 0.0410	0.0980 ± 0.0029	10.2479 ± 0.06960	0.6471 ± 0.0178	0.9947 ± 0.0122	34.8335 ± 0.1766	1.3318 ± 0.0246	0.5693 ± 0.0031
	TT(136–147)	0.4106 ± 0.0157^B^	0.9285 ± 0.0134^B^	2.7934 ± 0.0392^B^	0.0579 ± 0.0040	2.9274 ± 0.0518	0.0968 ± 0.0040	10.1267 ± 0.0874	0.6600 ± 0.0243	0.9804 ± 0.0166	34.6245 ± 0.2293	1.3495 ± 0.0323	0.5746 ± 0.0042
	*P*	<.0001^**^	<.0001^**^	0.0022^**^	0.8856	0.2126	0.6079	0.2572	0.7288	0.5650	0.4155	0.8199	0.2053
g. 146737879 T > G	GG(137–148)	0.4117 ± 0.0158^A^	0.9053 ± 0.0135^Aa^	2.7827 ± 0.0399^A^	0.0575 ± 0.0040	2.9852 ± 0.0514	0.0962 ± 0.0040	10.1224 ± 0.0876	0.6588 ± 0.0239	0.9809 ± 0.0167	34.7225 ± 0.2283	1.3232 ± 0.0319	0.5739 ± 0.0042
	GT(416–452)	0.5013 ± 0.0121^B^	0.9359 ± 0.0107^Ab^	2.8248 ± 0.0323^a^	0.0589 ± 0.0035	3.0364 ± 0.0412	0.0977 ± 0.0029	10.2698 ± 0.0696	0.6513 ± 0.0178	0.9975 ± 0.0122	34.8734 ± 0.1770	1.3154 ± 0.0246	0.5663 ± 0.0031
	TT(314–341)	0.5384 ± 0.0130^C^	0.9831 ± 0.0113^B^	2.8914 ± 0.0337^Bb^	0.0596 ± 0.0036	3.0380 ± 0.0437	0.0998 ± 0.0031	10.2327 ± 0.0735	0.6594 ± 0.0194	1.0019 ± 0.0132	34.7756 ± 0.1892	1.3229 ± 0.0266	0.5655 ± 0.0034
	*P*	<.0001^**^	<.0001^**^	0.0017^**^	0.7680	0.4448	0.6531	0.1295	0.8731	0.4325	0.6984	0.9269	0.0919
g. 146737916 T > C	CC(139–151)	0.3909 ± 0.0155^A^	0.9080 ± 0.0133^A^	2.8087 ± 0.0392^a^	0.0572 ± 0.0040	2.9481 ± 0.0512	0.0969 ± 0.0040	10.2239 ± 0.0870	0.6547 ± 0.0239	0.9769 ± 0.0165	34.6490 ± 0.2278	1.3256 ± 0.0320	0.5741 ± 0.0042
	TT(315–342)	0.5185 ± 0.0129^B^	0.9891 ± 0.0113^B^	2.9066 ± 0.0338^b^	0.0582 ± 0.0036	3.0094 ± 0.0435	0.1009 ± 0.0031	10.2947 ± 0.0734	0.6549 ± 0.0194	0.9981 ± 0.0131	34.7363 ± 0.1881	1.3300 ± 0.0266	0.5672 ± 0.0033
	CT(419–454)	0.4963 ± 0.0122^B^	0.9291 ± 0.0107^A^	2.8552 ± 0.0320	0.0580 ± 0.0035	3.0156 ± 0.0411	0.0983 ± 0.0029	10.3252 ± 0.0696	0.6464 ± 0.0180	0.9921 ± 0.0122	34.7903 ± 0.1759	1.3212 ± 0.0246	0.5683 ± 0.0031
	*P*	<.0001^**^	<.0001^**^	0.0088^**^	0.9406	0.2742	0.5637	0.3730	0.8589	0.4280	0.7790	0.9201	0.2206
g. 146737946C > T	CC(309–336)	0.5219 ± 0.0130^A^	1.0049 ± 0.0113^A^	2.8350 ± 0.0341^a^	0.0588 ± 0.0036	2.9994 ± 0.0439	0.1004 ± 0.0032	10.2241 ± 0.0738	0.6597 ± 0.0193	1.0016 ± 0.0134	34.7602 ± 0.1890	1.3334 ± 0.0264	0.5630 ± 0.0034
	TT(138–149)	0.4155 ± 0.0156^B^	0.9113 ± 0.0135^B^	2.7486 ± 0.0392^b^	0.0575 ± 0.0040	2.9507 ± 0.0512	0.0968 ± 0.0040	10.1050 ± 0.0876	0.6572 ± 0.0242	0.9796 ± 0.0165	34.6590 ± 0.2300	1.3427 ± 0.0324	0.5682 ± 0.0042
	CT(424–460)	0.4999 ± 0.0122^A^	0.9464 ± 0.0106^C^	2.7811 ± 0.0319	0.0582 ± 0.0035	3.0044 ± 0.0411	0.0981 ± 0.0029	10.2450 ± 0.0689	0.6447 ± 0.0179	0.9973 ± 0.0121	34.8888 ± 0.1760	1.3304 ± 0.0247	0.5650 ± 0.0031
	*P*	<.0001^**^	<.0001^**^	0.0151^*^	0.8961	0.4414	0.6268	0.1510	0.6382	0.3945	0.4662	0.9099	0.4202
g. 146738055G > A	AA(10–12)	0.6010 ± 0.0459^A^	1.0161 ± 0.0354	2.8684 ± 0.1023	0.0479 ± 0.0090	3.0416 ± 0.1363	0.0976 ± 0.0122	10.3654 ± 0.2293	0.6569 ± 0.0695	0.9913 ± 0.0486	35.1841 ± 0.6782	1.3472 ± 0.0940	0.5674 ± 0.0122
	AG(218–237)	0.5257 ± 0.0138^A^	0.9751 ± 0.0122^A^	2.7914 ± 0.0360	0.0584 ± 0.0037	2.9691 ± 0.0461	0.0968 ± 0.0035	10.1430 ± 0.0783	0.6599 ± 0.0210	0.9892 ± 0.0144	34.7921 ± 0.2040	1.3007 ± 0.0284	0.5638 ± 0.0036
	GG(650–700)	0.4368 ± 0.0115^B^	0.9454 ± 0.0102^B^	2.8074 ± 0.0310	0.0592 ± 0.0034	3.0454 ± 0.0394	0.1003 ± 0.0026	10.2445 ± 0.0669	0.6563 ± 0.0169	0.9931 ± 0.0115	34.7389 ± 0.1667	1.3298 ± 0.0234	0.5711 ± 0.0029
	*P*	<.0001^**^	0.0010^**^	0.6593	0.3930	0.0792	0.5412	0.1855	0.9801	0.9508	0.7698	0.4293	0.0622
**SNP**	Genotype (No.)	C17:1, %	C18:0, %	C18:1*cis*-9. %	C18index, %	C20:0, %	C14index, %	C16index, %	C17index, %	SFA, %	UFA, %	SFA/UFA	Total index, %
g. 146702957G > A	AA(62–73)	0.1943 ± 0.0042	13.8892 ± 0.1615	19.1043 ± 0.2147	57.6323 ± 0.4830	0.1666 ± 0.0031	6.4077 ± 0.2370	3.7684 ± 0.1055	25.6833 ± 0.3643	67.7650 ± 0.2901	30.4810 ± 0.2651	2.2673 ± 0.0383	27.6986 ± 0.2400
	GG(424–511)	0.1895 ± 0.0024	14.1758 ± 0.0849	19.1790 ± 0.1155	57.3457 ± 0.2687	0.1722 ± 0.0017^A^	6.1345 ± 0.1378	3.6753 ± 0.0635	24.9133 ± 0.2143	67.8726 ± 0.1595	30.4841 ± 0.1442	2.2689 ± 0.0209	27.6651 ± 0.1408^a^
	GA(367–395)	0.1894 ± 0.0026	14.0807 ± 0.0916	18.9544 ± 0.1220	56.9727 ± 0.2812	0.1656 ± 0.0018^B^	6.3010 ± 0.1449	3.6562 ± 0.0658	24.9476 ± 0.2258	68.0908 ± 0.1672	30.1838 ± 0.1525	2.2946 ± 0.0221	27.3392 ± 0.1492^b^
	*P*	0.4386	0.1580	0.1290	0.1783	0.0001^**^	0.2470	0.5211	0.0648	0.2479	0.0767	0.3992	0.0137^*^
g. 146704373A > G	AA(420–507)	0.1913 ± 0.0024	14.1259 ± 0.0853	19.2191 ± 0.1150	57.5363 ± 0.2686	0.1730 ± 0.0017^A^	6.0812 ± 0.1397	3.6613 ± 0.0633	24.8788 ± 0.2148	67.8406 ± 0.1588	30.4771 ± 0.1448	2.2695 ± 0.0210	27.5503 ± 0.1427^A^
	GG(62–73)	0.1968 ± 0.0042	13.8793 ± 0.1644	19.1654 ± 0.2165	57.9715 ± 0.4858	0.1683 ± 0.0030	6.3322 ± 0.2387	3.7324 ± 0.1072	25.6137 ± 0.3613	67.7508 ± 0.2936	30.5383 ± 0.2663	2.2679 ± 0.0387	27.5179 ± 0.2397
	GA(290–361)	0.1909 ± 0.0026	13.9943 ± 0.0926	18.9787 ± 0.1240	57.1583 ± 0.2841	0.1660 ± 0.0018^B^	6.2187 ± 0.1449	3.6303 ± 0.0667	24.8785 ± 0.2281	68.1000 ± 0.1698	30.1883 ± 0.1544	2.2966 ± 0.0224	27.1837 ± 0.1520^B^
	*P*	0.3096	0.1494	0.0995	0.1186	<.0001^**^	0.3517	0.5611	0.0732	0.1635	0.0790	0.3715	0.0065^**^
g. 146704618A > G	AA(392–475)	0.1929 ± 0.0025	14.1934 ± 0.0867^a^	19.3545 ± 0.1167^a^	57.3032 ± 0.2720	0.1721 ± 0.0017^A^	6.1572 ± 0.1406	3.6288 ± 0.0640	24.6723 ± 0.2156	67.9068 ± 0.1614	30.5624 ± 0.1480	2.2704 ± 0.0212	27.6612 ± 0.1445^a^
	GG(69–84)	0.1980 ± 0.0039	13.8955 ± 0.1515	19.2130 ± 0.2015	57.7299 ± 0.4541	0.1661 ± 0.0029	6.3577 ± 0.2214	3.6981 ± 0.1002	25.3871 ± 0.3399	67.7817 ± 0.2744	30.6622 ± 0.2488	2.2769 ± 0.0362	27.7215 ± 0.2257
	GA(324–396)	0.1916 ± 0.0025	13.9823 ± 0.0885^b^	19.0297 ± 0.1199^b^	57.2219 ± 0.2775	0.1668 ± 0.0018^B^	6.3184 ± 0.1421	3.6029 ± 0.0647	24.6942 ± 0.2223	68.1314 ± 0.1646	30.2507 ± 0.1491	2.3049 ± 0.0217	27.3551 ± 0.1486^b^
	*P*	0.2123	0.0182^*^	0.0137^*^	0.4975	0.0024^**^	0.3292	0.5736	0.0623	0.2011	0.0386^*^	0.2066	0.0173^*^
g. 146704699G > A	AA(92–102)	0.1903 ± 0.0036	13.8701 ± 0.1379^b^	19.3065 ± 0.1844	57.8611 ± 0.4127	0.1715 ± 0.0025	6.2255 ± 0.2057	3.5700 ± 0.0924	24.3088 ± 0.3144^A^	67.8410 ± 0.2477	30.3455 ± 0.2268	2.2693 ± 0.0330	27.7433 ± 0.2080
	AG(324–443)	0.1886 ± 0.0025	14.2179 ± 0.0895^Aa^	19.1440 ± 0.1202	57.1301 ± 0.2779^a^	0.1695 ± 0.0017	6.1203 ± 0.1428	3.6496 ± 0.0646	24.5937 ± 0.2213^A^	68.0257 ± 0.1649	30.3321 ± 0.1507	2.2872 ± 0.0217	27.3897 ± 0.1458
	GG(365–443)	0.1935 ± 0.0025	13.9484 ± 0.0895^B^	19.2363 ± 0.1189	57.7454 ± 0.2775^b^	0.1683 ± 0.0017	6.2892 ± 0.1427	3.7083 ± 0.0653	25.1974 ± 0.2243^B^	67.8451 ± 0.1655	30.4898 ± 0.1492	2.2789 ± 0.0219	27.6124 ± 0.1479
	*P*	0.0659	0.0020^**^	0.5640	0.0249^*^	0.4108	0.3721	0.2267	0.0006^**^	0.4504	0.5095	0.8274	0.0692
g. 146705692G > A	AA(7–9)	0.1733 ± 0.0099	14.3208 ± 0.3965	19.6663 ± 0.5313	57.2586 ± 1.1637	0.1951 ± 0.0077^A^	6.0520 ± 0.5535	3.4819 ± 0.2506	23.4125 ± 0.8489	68.1513 ± 0.7081	30.5992 ± 0.6477	2.2889 ± 0.0942	27.5978 ± 0.5625
	GG(575–694)	0.1888 ± 0.0023	14.1146 ± 0.0794	19.2113 ± 0.1066^A^	57.3378 ± 0.2523	0.1706 ± 0.0016^B^	6.3397 ± 0.1305	3.6825 ± 0.0560	25.0231 ± 0.2052	67.9010 ± 0.1488	30.4947 ± 0.1354	2.2644 ± 0.0197	27.5684 ± 0.1365^A^
	GA(200–250)	0.1887 ± 0.0028	14.0965 ± 0.1040	18.8330 ± 0.1399^B^	56.9865 ± 0.3164	0.1645 ± 0.0020^C^	6.1097 ± 0.1620	3.6635 ± 0.0723	25.0126 ± 0.2497	68.2087 ± 0.1901	30.1774 ± 0.1714	2.2906 ± 0.0251	27.1387 ± 0.1658^B^
	*P*	0.2854	0.8464	0.0042^**^	0.4179	<.0001^**^	0.1835	0.6902	0.1576	0.159	0.0946	0.4718	0.0034^**^
g. 146725085 T > C	CC(397–486)	0.1909 ± 0.0025	14.0468 ± 0.0870	19.0157 ± 0.1164	57.2455 ± 0.2729	0.1677 ± 0.0017^A^	6.1641 ± 0.1391	3.6277 ± 0.0641	24.8455 ± 0.2162	68.0279 ± 0.1619	30.2848 ± 0.1468	2.2931 ± 0.0213	27.3956 ± 0.1473^a^
	CT(311–378)	0.1942 ± 0.0026	14.0184 ± 0.0911	19.2591 ± 0.1234	57.6639 ± 0.2843	0.1712 ± 0.0018	6.2935 ± 0.1457	3.7375 ± 0.0663	25.2682 ± 0.2286	67.7874 ± 0.1695	30.5957 ± 0.1552	2.2615 ± 0.0222	27.6832 ± 0.1486^b^
	TT(58–70)	0.1995 ± 0.0042	14.0374 ± 0.1585	19.4598 ± 0.2118	57.8594 ± 0.4752	0.1768 ± 0.0030^B^	6.5092 ± 0.2316	3.5625 ± 0.1057	25.4392 ± 0.3615	67.6933 ± 0.2875	30.6268 ± 0.2630	2.2696 ± 0.0381	27.8078 ± 0.2388
	*P*	0.0502	0.9442	0.0227^*^	0.1449	0.0026^**^	0.2133	0.0520	0.0294^*^	0.1896	0.0542	0.2723	0.0207^*^
g. 146726096A > G	AA(672–805)	0.1894 ± 0.0023^A^	14.0391 ± 0.0782^a^	19.0281 ± 0.1066^A^	57.3355 ± 0.2478	0.169 ± 0.0015^A^	6.1731 ± 0.1295	3.6343 ± 0.0594	24.7419 ± 0.1997^A^	68.0865 ± 0.1467^A^	30.2985 ± 0.1327^Aa^	2.2925 ± 0.0193^A^	27.4019 ± 0.1349^A^
	AG(107–145)	0.2020 ± 0.0032^B^	14.3151 ± 0.1199^b^	19.6764 ± 0.1610^B^	57.6605 ± 0.3664	0.1673 ± 0.0024^A^	6.3518 ± 0.1821	3.6312 ± 0.0822	25.5638 ± 0.2780^B^	67.3379 ± 0.2192^Bb^	31.0017 ± 0.1996^B^	2.2118 ± 0.0288^Bb^	28.0468 ± 0.1811^Bb^
	GG(4–5)	0.1718 ± 0.0133	14.6475 ± 0.5272	18.4707 ± 0.7012	55.4830 ± 1.5514	0.1989 ± 0.0100^B^	5.1590 ± 0.7400	3.3763 ± 0.3321	23.7060 ± 1.1232	70.1075 ± 0.9410^a^	28.1258 ± 0.8622^Ab^	2.5679 ± 0.1243^a^	25.9007 ± 0.7434^a^
	*P*	<.0001^**^	0.0246^*^	<.0001^**^	0.2737	0.0069^**^	0.1794	0.7344	0.0011^**^	<.0001^**^	<.0001^**^	0.0005^**^	<.0001^**^
g. 146729107A > C	AA(157–183)	0.1904 ± 0.0031	14.0091 ± 0.1119	19.2419 ± 0.1482	57.4468 ± 0.3385	0.1745 ± 0.0021^A^	6.3828 ± 0.1703	3.6593 ± 0.0779	25.1709 ± 0.2648	67.8911 ± 0.2031	30.4868 ± 0.185	2.2755 ± 0.0266	27.7110 ± 0.1753
	CC(219–259)	0.1883 ± 0.0028	14.1061 ± 0.1012	19.1574 ± 0.1368	57.4101 ± 0.3144	0.1676 ± 0.0020^C^	6.0951 ± 0.1568	3.6834 ± 0.0720	24.7129 ± 0.2420	67.7963 ± 0.1872	30.5267 ± 0.1705	2.2672 ± 0.0248	27.5823 ± 0.1642
	CA(395–494)	0.1894 ± 0.0025	13.9829 ± 0.0864	19.1133 ± 0.1161	57.5354 ± 0.2712	0.1703 ± 0.0017	6.0679 ± 0.1391	3.6817 ± 0.0634	24.9844 ± 0.2181	67.9785 ± 0.1603	30.3797 ± 0.1454	2.2879 ± 0.0213	27.5050 ± 0.1431
	*P*	0.7788	0.4157	0.6456	0.8887	0.0072^**^	0.0883	0.9350	0.1738	0.5431	0.5871	0.6297	0.3631
g. 146735090G > T	GG(476–569)	0.1922 ± 0.0024	14.0441 ± 0.0833	19.0282 ± 0.1124	57.3698 ± 0.2622	0.1712 ± 0.0016	6.0957 ± 0.1366	3.6985 ± 0.0616	24.8158 ± 0.2105	68.0759 ± 0.1559	30.3923 ± 0.1411	2.2819 ± 0.0204	27.5196 ± 0.1422
	GT(284–351)	0.1934 ± 0.0026	14.1213 ± 0.0926	19.0764 ± 0.1241	57.0423 ± 0.2871	0.1697 ± 0.0018	6.2102 ± 0.146	3.6004 ± 0.0664	24.8500 ± 0.2284	67.9829 ± 0.1706	30.3837 ± 0.1551	2.2809 ± 0.0226	27.4330 ± 0.1490
	TT(20–31)	0.1984 ± 0.0058	14.3172 ± 0.2303	18.8872 ± 0.3054	56.3973 ± 0.6774	0.1786 ± 0.0049	6.0045 ± 0.3251	3.6534 ± 0.1493	24.9340 ± 0.5123	68.2537 ± 0.4099	30.2155 ± 0.3762	2.3215 ± 0.0547	27.2316 ± 0.3293
	*P*	0.4841	0.3646	0.7827	0.1741	0.1494	0.5571	0.1695	0.9587	0.7056	0.8890	0.7477	0.5447
g. 146737188C > T	CC(284–344)	0.1937 ± 0.0026^b^	14.0802 ± 0.0941	19.0166 ± 0.1268	57.2315 ± 0.2934	0.1716 ± 0.0018	6.1741 ± 0.1492	3.6909 ± 0.0677	25.3794 ± 0.2296	68.0396 ± 0.1746	30.3617 ± 0.1578	2.2880 ± 0.0228	27.4624 ± 0.1555
	CT(361–448)	0.1877 ± 0.0025^Aa^	14.1547 ± 0.0872	18.9851 ± 0.1163	57.0650 ± 0.2710	0.1689 ± 0.0017	6.1248 ± 0.1392	3.6688 ± 0.0638	24.9450 ± 0.2165	68.0867 ± 0.1608	30.2470 ± 0.1460	2.2958 ± 0.0214	27.4299 ± 0.1436^a^
	TT(127–148)	0.1961 ± 0.0032^B^	14.1171 ± 0.1204	19.3227 ± 0.1595	57.5732 ± 0.3627	0.1687 ± 0.0022	6.1684 ± 0.1817	3.7299 ± 0.0820	25.2577 ± 0.2793	67.6431 ± 0.2173	30.6818 ± 0.1987	2.2482 ± 0.0291	27.8433 ± 0.1864^b^
	*P*	0.0017^**^	0.6908	0.0814	0.3152	0.1887	0.9103	0.6926	0.0529	0.0888	0.0682	0.2209	0.0318^*^
g. 146737545G > A	AA(128–148)	0.1945 ± 0.0032^a^	14.0514 ± 0.1204	19.4701 ± 0.1601	57.9106 ± 0.3655	0.1696 ± 0.0023	6.2266 ± 0.1817	3.6997 ± 0.0817	24.9182 ± 0.2810	67.4893 ± 0.2187^a^	30.7602 ± 0.1992	2.2208 ± 0.0288	27.9350 ± 0.1870^a^
	GG(283–343)	0.1931 ± 0.0026^A^	14.0176 ± 0.0944	19.1472 ± 0.1258	57.4943 ± 0.2900	0.1711 ± 0.0018	6.2523 ± 0.1485	3.6694 ± 0.0677	25.0728 ± 0.2302	67.9662 ± 0.1728	30.3993 ± 0.1579	2.2747 ± 0.0230	27.5125 ± 0.1534^b^
	GA(368–455)	0.1866 ± 0.0025^Bb^	14.0964 ± 0.0863	19.1421 ± 0.1158	57.3574 ± 0.268	0.1686 ± 0.0017	6.1186 ± 0.1391	3.6470 ± 0.0635	24.6877 ± 0.2175	68.0117 ± 0.1589^b^	30.3193 ± 0.1449	2.2817 ± 0.0210	27.5136 ± 0.1426^b^
	*P*	0.0017^**^	0.6564	0.0816	0.2603	0.3128	0.5090	0.7494	0.1064	0.0334^*^	0.0622	0.0756	0.0232^*^
g. 146737748 T > C	CC(130–151)	0.1979 ± 0.0032^a^	14.0913 ± 0.1188	19.3639 ± 0.1597	57.7773 ± 0.3626	0.1711 ± 0.0022	6.2361 ± 0.1823	3.6841 ± 0.0805	24.9868 ± 0.2789	67.5538 ± 0.2170	30.6795 ± 0.1968	2.2365 ± 0.0285	27.8452 ± 0.1856^a^
	CT(366–455)	0.1899 ± 0.0025^b^	14.0907 ± 0.0871	19.0535 ± 0.1157	57.2339 ± 0.2677	0.1698 ± 0.0017	6.1569 ± 0.1387	3.6787 ± 0.0634	24.8028 ± 0.2166	68.0054 ± 0.1596	30.2417 ± 0.1456	2.2852 ± 0.0211	27.4570 ± 0.1426^b^
	TT(281–341)	0.1954 ± 0.0026^a^	14.0418 ± 0.0941	19.0741 ± 0.1267	57.2531 ± 0.2940	0.1725 ± 0.0018	6.2670 ± 0.1472	3.6871 ± 0.0681	25.1846 ± 0.2305	68.0076 ± 0.1746	30.3295 ± 0.1583	2.2865 ± 0.0230	27.4242 ± 0.1548^b^
	*P*	0.0043^**^	0.8370	0.1110	0.2416	0.2563	0.6416	0.9876	0.1187	0.0646	0.0634	0.1604	0.0294^*^
g. 146737849C > T	CC(280–340)	0.1942 ± 0.0026^b^	14.0479 ± 0.0944	19.0461 ± 0.1275	57.2827 ± 0.2941	0.1715 ± 0.0018	6.2705 ± 0.1497	3.6784 ± 0.0679	25.2872 ± 0.2299^a^	68.0036 ± 0.1752	30.2563 ± 0.1585	2.2749 ± 0.0231	27.3959 ± 0.1549
	CT(369–458)	0.1884 ± 0.0025^Aa^	14.1343 ± 0.0867	19.0311 ± 0.1157	57.1311 ± 0.2686	0.1692 ± 0.0017	6.1599 ± 0.1381	3.6433 ± 0.0634	24.8370 ± 0.2170^b^	68.1156 ± 0.1595^a^	30.1612 ± 0.1454	2.2852 ± 0.0211	27.3368 ± 0.1429^a^
	TT(126–147)	0.1968 ± 0.0032^B^	14.1515 ± 0.1209	19.3218 ± 0.1608	57.6388 ± 0.3647	0.1698 ± 0.0023	6.2904 ± 0.1813	3.7417 ± 0.0821	25.1958 ± 0.2806	67.599 ± 0.2189^b^	30.5937 ± 0.1985	2.2359 ± 0.0289	27.7909 ± 0.1867^b^
	*P*	0.0022^**^	0.5467	0.1496	0.3216	0.3580	0.5645	0.3874	0.0389^*^	0.0434^*^	0.0724	0.2010	0.0181^*^
g. 146737879 T > G	GG(127–148)	0.1950 ± 0.0032^A^	14.1331 ± 0.1199	19.2864 ± 0.1606	57.5884 ± 0.3617	0.1668 ± 0.0023	6.1986 ± 0.1806	3.6782 ± 0.0813	25.1214 ± 0.2807	67.5294 ± 0.2172	30.6096 ± 0.1983	2.2448 ± 0.0290	27.8407 ± 0.1859^a^
	GT(363–452)	0.1860 ± 0.0025^B^	14.0937 ± 0.0864	18.9996 ± 0.1170	57.1175 ± 0.2712	0.1662 ± 0.0017	6.0816 ± 0.1386	3.6252 ± 0.0634	24.9443 ± 0.2164	67.9798 ± 0.1605	30.2380 ± 0.1464	2.2917 ± 0.0212	27.4284 ± 0.1438^b^
	TT(281–341)	0.1926 ± 0.0026^A^	14.0683 ± 0.0950	18.9898 ± 0.1260	57.2233 ± 0.2916	0.1694 ± 0.0018	6.2251 ± 0.1478	3.6573 ± 0.0683	25.3307 ± 0.2299	67.9566 ± 0.1740	30.2822 ± 0.1578	2.2935 ± 0.0228	27.4250 ± 0.1552^b^
	*P*	0.0007^**^	0.8640	0.1380	0.3760	0.1390	0.4621	0.7092	0.1116	0.0763	0.1374	0.1878	0.0258^*^
g. 146737916 T > C	CC(130–151)	0.1958 ± 0.0031^a^	14.0216 ± 0.1184	19.4421 ± 0.1594	57.8855 ± 0.3621	0.1702 ± 0.0023	6.2201 ± 0.1802	3.675 ± 0.0810	25.1146 ± 0.2768	67.5382 ± 0.2176	30.7478 ± 0.1974	2.2388 ± 0.0284	27.8821 ± 0.1855^a^
	TT(282–342)	0.1941 ± 0.0026^A^	13.9730 ± 0.0943	19.1122 ± 0.1261	57.3779 ± 0.2917	0.1720 ± 0.0018	6.2657 ± 0.1487	3.6778 ± 0.0683	25.3034 ± 0.2313	67.9870 ± 0.1741	30.3957 ± 0.1581	2.2897 ± 0.0229	27.4459 ± 0.1536^b^
	CT(366–454)	0.1876 ± 0.0025^Bb^	14.0312 ± 0.0863	19.1531 ± 0.1157	57.3551 ± 0.2685	0.1692 ± 0.0017	6.1427 ± 0.1386	3.6574 ± 0.0634	24.9351 ± 0.2172	67.9939 ± 0.1593	30.3340 ± 0.1453	2.2877 ± 0.0211	27.4913 ± 0.1430^b^
	*P*	0.0014^**^	0.7903	0.1021	0.2644	0.2229	0.5851	0.9227	0.1358	0.0673	0.0831	0.1542	0.0260^*^
g. 146737946C > T	CC(276–353)	0.1934 ± 0.0026^a^	14.0356 ± 0.0944	19.0648 ± 0.1269	57.3541 ± 0.2931	0.1703 ± 0.0018	6.2878 ± 0.1493	3.6822 ± 0.0677	25.2621 ± 0.2307	67.9820 ± 0.1743	30.4044 ± 0.1588	2.2781 ± 0.0231	27.4462 ± 0.1556^b^
	TT(129–149)	0.1956 ± 0.0032^A^	14.0929 ± 0.1198	19.3950 ± 0.1605	57.7406 ± 0.3642	0.1687 ± 0.0022	6.2543 ± 0.1810	3.7333 ± 0.0830	24.8679 ± 0.2786	67.5357 ± 0.2182	30.7658 ± 0.1999	2.2291 ± 0.0290	27.9048 ± 0.1867^Aa^
	CT(372–460)	0.1872 ± 0.0025^Bb^	14.1087 ± 0.0864	19.0634 ± 0.1159	57.2223 ± 0.2683	0.1683 ± 0.0017	6.1445 ± 0.1379	3.6631 ± 0.0633	24.8255 ± 0.2163	68.0108 ± 0.1592	30.3468 ± 0.1450	2.2787 ± 0.0210	27.4151 ± 0.1428^B^
	*P*	0.0015^**^	0.6954	0.0742	0.3013	0.4732	0.4699	0.6201	0.0533	0.0567	0.0783	0.1645	0.0073^**^
g. 146738055G > A	AA(8–12)	0.2017 ± 0.0090	13.9011 ± 0.3614	18.0083 ± 0.4790	55.7325 ± 1.0632	0.1754 ± 0.0077	5.8460 ± 0.5058	3.6632 ± 0.2374	24.8286 ± 0.8041	69.0285 ± 0.6442	29.7689 ± 0.5866	2.3942 ± 0.0853	26.4994 ± 0.5087^a^
	AG(195–237)	0.1868 ± 0.0029^a^	14.1406 ± 0.1041	18.9628 ± 0.1381	56.8884 ± 0.3155	0.1674 ± 0.0020	6.2627 ± 0.1586	3.5945 ± 0.0726	24.4635 ± 0.2501^a^	68.2252 ± 0.1883	30.2599 ± 0.1723	2.2993 ± 0.0251	27.2869 ± 0.1638^b^
	GG(578–702)	0.1929 ± 0.0023^b^	14.1562 ± 0.0803	19.1422 ± 0.1083	57.3907 ± 0.2534	0.1702 ± 0.0016	6.1662 ± 0.1317	3.6781 ± 0.0602	24.9766 ± 0.2047^b^	68.0300 ± 0.1500	30.5244 ± 0.1358	2.2729 ± 0.0197	27.5622 ± 0.1357
	*P*	0.0149^*^	0.7729	0.0258^*^	0.0632	0.2047	0.5865	0.3532	0.0353^*^	0.1638	0.1078	0.1927	0.0146^*^

Note: *LSM* least square mean. *SE* standard error. *P* indicates the significances of the association analysis between each SNP and milk fatty acid traits. *P* is the raw value. *: *P* < 0.05. **: *P* < 0.01. Different letter (small letters: *P* < 0.05; capital letters: *P* < 0.01) superscripts indicate significant differences among the genotypes. The number in the brackets represents the number of cows for the corresponding genotypes

Further, the additive (a), dominant (d), and allele substitution effects (α) of the 17 SNPs on each kind of fatty acid were calculated. Results showed that the 17 SNPs exhibited significant additive, dominant, and allele substitution effects on C6:0, C8:0, C10:0, C14:0, C16:0, C16:1, C17:0, C18:0, C18:1*cis*-9, C18index, C20:0, C14index, C16index, C17index, SFA, UFA, and total index (Table S2; *P* < 0.05). For C11:0, C12:0, C13:0, C14:1 and C15:0, none of significant additive, dominant, and allele substitution effects was found (*P* > 0.05).

Also, association analysis on two haplotype blocks with 24 milk FAs was performed (Table 3). The haplotype blcok1 was significantly associated with C6:0, C8:0, C10:0, C14:0, C18:0, C20:0, C17index and total index (*P* < 0.0001–0.0245), and the block 2 was strongly associated with C6:0, C8:0, C10:0, C14:0, C18:0, C17:1, C18:1*cis*-9, C18index, C20:0, C16index, C17index, SFA, UFA and total index (*P* < 0.0001–0.0498; Table 3). While, no significant association was found for C11:0, C12:0, C13:0, C14:1, C15:0, C16:0, C16:1, C14index and SFA/UFA (*P* > 0.05).

**Table 3:** Association between haplotype blocks in *AGPAT3* and milk fatty acid traits in Chinese Holstein cows (LSM ± SE)

Haplotype block	Haplotype combination (No.)	C6:0, %	C8:0, %	C10:0, %	C11:0, %	C12:0, %	C13:0, %	C14:0, %	C14:1, %	C15:0, %	C16:0, %	C16:1, %	C17:0, %
Block 1	H1H1(121–130)	0.3692 ± 0.0166^A^	0.8989 ± 0.0140^ACa^	2.7932 ± 0.0413^a^	0.0579 ± 0.0041	2.9643 ± 0.0538	0.1020 ± 0.0043	10.1573 ± 0.0917	0.6359 ± 0.0253	0.9978 ± 0.0175	34.9164 ± 0.2400	1.3602 ± 0.0336	0.5739 ± 0.0044
	H1H2(218–240)	0.4422 ± 0.0142^B^	0.8923 ± 0.0122^Aa^	2.7610 ± 0.0367^A^	0.0569 ± 0.0038	2.9761 ± 0.0470	0.0983 ± 0.0035	10.2724 ± 0.0804	0.6296 ± 0.0213	0.9938 ± 0.0149	34.8598 ± 0.2082	1.3269 ± 0.0292	0.5767 ± 0.0037
	H1H3(191–204)	0.5563 ± 0.0146^C^	1.0162 ± 0.0128^Bb^	2.9065 ± 0.0373^Bb^	0.0596 ± 0.0039	3.0334 ± 0.0487	0.1005 ± 0.0037	10.3997 ± 0.0839^a^	0.6786 ± 0.0225	1.0068 ± 0.0153	34.7483 ± 0.2175	1.3176 ± 0.0303	0.5656 ± 0.0039
	H2H2(98–103)	0.4381 ± 0.0175^B^	0.9044 ± 0.0152^ACDac^	2.8120 ± 0.0436	0.0594 ± 0.0043	2.9978 ± 0.0575	0.0987 ± 0.0046	10.3001 ± 0.0986	0.6575 ± 0.0274	0.9853 ± 0.0190	34.7264 ± 0.2591	1.2820 ± 0.0363	0.5686 ± 0.0048
	H2H3(136–150)	0.4610 ± 0.0157^B^	0.9441 ± 0.0134^CDcd^	2.7551 ± 0.0400^A^	0.0575 ± 0.0040	2.9299 ± 0.0521	0.0956 ± 0.0041	10.1961 ± 0.0869	0.6483 ± 0.0243	0.9835 ± 0.0166	35.0655 ± 0.2324	1.3081 ± 0.0326	0.5694 ± 0.0042
	H3H3(66–72)	0.5532 ± 0.0206^C^	0.9642 ± 0.0177^BDd^	2.8116 ± 0.0513	0.0597 ± 0.0048	3.0030 ± 0.0657	0.0978 ± 0.0056	10.0632 ± 0.1132^b^	0.6629 ± 0.0329	0.9895 ± 0.0227	34.8421 ± 0.3116	1.3749 ± 0.0428	0.5689 ± 0.0056
	*P*	<.0001**	<.0001**	0.0001**	0.9269	0.4138	0.8544	0.0105*	0.3696	0.8195	0.8036	0.3084	0.0962
Block 2	H1H2(126–139)	0.5694 ± 0.0167^Aa^	0.9932 ± 0.0145^Aa^	2.8297 ± 0.0425^a^	0.0580 ± 0.0042	3.0485 ± 0.0549	0.0962 ± 0.0043	10.1137 ± 0.0946	0.6361 ± 0.0253	0.9918 ± 0.0176	34.9810 ± 0.2481	1.3447 ± 0.0344	0.5703 ± 0.0045
	H1H3(56–62)	0.6103 ± 0.0213^Da^	1.0604 ± 0.0180^B^	2.8139 ± 0.0519	0.0575 ± 0.0050	2.9637 ± 0.0688	0.0984 ± 0.0057	9.9868 ± 0.1182	0.6793 ± 0.0335	0.9893 ± 0.0231	34.7801 ± 0.3163	1.3754 ± 0.0439	0.5671 ± 0.0058
	H1H4(52–55)	0.4357 ± 0.0224^Bbc^	0.9280 ± 0.0192^ACd^	2.8422 ± 0.0552	0.0631 ± 0.0053	3.0676 ± 0.0718	0.1029 ± 0.0061	10.3528 ± 0.1232	0.6664 ± 0.0353	1.0077 ± 0.0243	34.2739 ± 0.3353	1.4103 ± 0.0465	0.5652 ± 0.0062
	H2H2(138–149)	0.3787 ± 0.0166^Cbe^	0.8735 ± 0.0140^Cde^	2.7289 ± 0.0417	0.0579 ± 0.0042	2.9456 ± 0.0546	0.0967 ± 0.0042	10.0997 ± 0.0927	0.6586 ± 0.0250	0.9760 ± 0.0171	34.7218 ± 0.2397	1.3455 ± 0.0335	0.5744 ± 0.0043
	H2H3(95–102)	0.5078 ± 0.0181^Ac^	0.8930 ± 0.0155^Cbd^	2.6966 ± 0.0455	0.0611 ± 0.0045	2.9215 ± 0.0594	0.0950 ± 0.0048	10.0970 ± 0.1008	0.6550 ± 0.0283	0.9821 ± 0.0192	35.2877 ± 0.2714	1.3358 ± 0.0376	0.5593 ± 0.0049
	H2H4(96–107)	0.4629 ± 0.0187^Fc^	0.8165 ± 0.0155^Dc^	2.6801 ± 0.0452^b^	0.0580 ± 0.0045	2.9780 ± 0.0594	0.1005 ± 0.0048	10.3642 ± 0.1024	0.6403 ± 0.0284	0.9990 ± 0.0193	34.9866 ± 0.2709	1.3292 ± 0.0373	0.5769 ± 0.0048
	H2H5(63–68)	0.3384 ± 0.0204^Ede^	0.8526 ± 0.0179^Cbce^	2.7312 ± 0.0510	0.0575 ± 0.0049	2.9783 ± 0.0671	0.0982 ± 0.0056	10.0688 ± 0.1129	0.6521 ± 0.0319	0.9807 ± 0.0223	34.6563 ± 0.3081	1.4043 ± 0.0426	0.5783 ± 0.0056
	*P*	<.0001**	<.0001**	0.0013**	0.8887	0.2555	0.9225	0.0127*	0.9124	0.8845	0.1205	0.4760	0.0193*
Haplotype block	Haplotype combination (No.)	C17:1, %	C18:0, %	C18:1*cis*-9, %	C18index, %	C20:0, %	C14index, %	C16index, %	C17index, %	SFA, %	UFA, %	SFA/UFA	Total index, %
Block 1	H1H1(105–130)	0.1992 ± 0.0034	14.1901 ± 0.1259	19.3463 ± 0.1683	57.4699 ± 0.3816	0.1791 ± 0.0025^Aad^	6.0996 ± 0.1885	3.7792 ± 0.0860	25.0181 ± 0.2938	67.7674 ± 0.2297	30.6829 ± 0.2089	2.2565 ± 0.0304	27.6878 ± 0.1931^a^
	H1H2(193–241)	0.1904 ± 0.0029	14.3563 ± 0.1050^a^	19.1954 ± 0.1414	56.9474 ± 0.3225	0.1710 ± 0.0020^ABc^	5.9684 ± 0.1634	3.6878 ± 0.0747	24.6437 ± 0.2483	67.9734 ± 0.1925	30.4159 ± 0.1766	2.2675 ± 0.0255	27.5081 ± 0.1649
	H1H3(167–204)	0.1926 ± 0.0030	13.9560 ± 0.1103^b^	19.0385 ± 0.1501	57.3701 ± 0.3377	0.1635 ± 0.0022^Bb^	6.3161 ± 0.1697	3.6750 ± 0.0774	24.9275 ± 0.2620	68.0604 ± 0.2014	30.1895 ± 0.1868	2.2838 ± 0.0269	27.4224 ± 0.1743
	H2H2(93–103)	0.1912 ± 0.0036	14.0036 ± 0.1391	19.2225 ± 0.1833	57.5431 ± 0.4143	0.1731 ± 0.0025^Aac^	6.1465 ± 0.2072	3.5740 ± 0.0928	24.2275 ± 0.3182^a^	67.9786 ± 0.2486	30.3912 ± 0.2271	2.2608 ± 0.0330	27.6407 ± 0.2079
	H2H3(119–150)	0.1922 ± 0.0032	14.2354 ± 0.1206	18.8881 ± 0.1604	56.5368 ± 0.3665	0.1697 ± 0.0023^Abc^	6.0705 ± 0.1814	3.6158 ± 0.0830	24.3598 ± 0.2842^a^	68.2035 ± 0.2194	30.1933 ± 0.1991	2.2977 ± 0.0289	27.0443 ± 0.1860^b^
	H3H3(61–72)	0.2002 ± 0.0043	13.8767 ± 0.1663	19.1521 ± 0.2201	57.8332 ± 0.4902	0.1669 ± 0.0031^Bbc^	6.3488 ± 0.2396	3.8032 ± 0.1081	25.6261 ± 0.3649^b^	67.7284 ± 0.2962	30.6459 ± 0.2693	2.2496 ± 0.0391	27.5612 ± 0.2436
	*P*	0.0513	0.0051**	0.214	0.0649	<.0001**	0.3560	0.2434	0.0053**	0.5462	0.1757	0.7634	0.0245*
Block 2	H1H2(117–139)	0.1873 ± 0.0034^A^	14.1418 ± 0.1267	19.0344 ± 0.1692	57.4628 ± 0.3893	0.1705 ± 0.0024	6.1665 ± 0.1940	3.6796 ± 0.0878	24.8359 ± 0.2983^a^	67.9941 ± 0.2332	30.3523 ± 0.2134	2.2848 ± 0.0307	27.5466 ± 0.1948
	H1H3(59–62)	0.1908 ± 0.0043	14.0962 ± 0.1695	19.0365 ± 0.2254	57.1552 ± 0.5004	0.1698 ± 0.0030	6.6035 ± 0.2487	3.7758 ± 0.1121	25.4165 ± 0.3779	67.9988 ± 0.3013	30.2962 ± 0.2735	2.2858 ± 0.0404	27.5735 ± 0.2497
	H1H4(44–55)	0.1991 ± 0.0046	13.7531 ± 0.1790	19.3446 ± 0.2403	58.2375 ± 0.5405	0.1771 ± 0.0035^A^	6.2159 ± 0.2621	3.9414 ± 0.1180	25.5598 ± 0.4005	67.3613 ± 0.3257	30.8744 ± 0.2977	2.2085 ± 0.0432	27.8206 ± 0.2639
	H2H2(128–149)	0.1973 ± 0.0034	14.1252 ± 0.1253	19.4270 ± 0.166^a^	57.7791 ± 0.3817	0.1679 ± 0.0024	6.2908 ± 0.1912	3.6936 ± 0.0856	25.0985 ± 0.2912	67.4990 ± 0.2285	30.7484 ± 0.2092^a^	2.2254 ± 0.0299	27.8762 ± 0.1924^A^
	H2H3(80–102)	0.1877 ± 0.0037^A^	14.0980 ± 0.1432	18.8617 ± 0.1897	56.6882 ± 0.4257	0.1714 ± 0.0028	6.3426 ± 0.2113	3.6030 ± 0.0958	24.7605 ± 0.3257^a^	68.2658 ± 0.2556	29.8952 ± 0.2352^b^	2.3174 ± 0.0344	27.0508 ± 0.2145^B^
	H2H4(88–107)	0.1877 ± 0.0038^a^	14.3357 ± 0.1411	18.7635 ± 0.1887^b^	56.6694 ± 0.4235	0.1622 ± 0.0028^B^	6.0775 ± 0.2131	3.6211 ± 0.0957	24.6392 ± 0.3234^a^	68.1996 ± 0.2544	30.0572 ± 0.2341	2.3011 ± 0.0339	27.0983 ± 0.2132^Ca^
	H2H5(50–69)	0.2051 ± 0.0042^Bb^	14.3662 ± 0.1625	19.5612 ± 0.2164^a^	57.5823 ± 0.4896	0.1644 ± 0.0033	6.1397 ± 0.2402	3.8763 ± 0.1080	25.9925 ± 0.368^b^	67.4171 ± 0.2947	30.8806 ± 0.2665^a^	2.2230 ± 0.0391	28.0083 ± 0.2341^ACb^
	*P*	<.0001**	0.0798	0.0036**	0.0345*	0.0020**	0.5789	0.0498*	0.0075**	0.0096**	0.0015**	0.0574	0.0001**

Note: *LSM* least square mean. *SE* standard error. *P* indicates the significances of the association analysis between the haplotype block and milk fatty acid traits. *P* is the raw value. ^*^: *P* < 0.05. ^**^: *P* < 0.01. Different letter (small letters: *P* < 0.05; capital letters: *P* < 0.01) superscripts indicate significant differences among the haplotype combinations. The number in the brackets represents the number of cows for the corresponding haplotype combination

### Prediction of TFBSs changing caused by the SNPs in 5′ regulatory region

By performing Genomatix software suite v3.9, it was predicted that that four SNPs in the 5′ regulatory region of *AGPAT3* gene, g.146702957G > A, g.146704373A > G, g.146704618A > G and g.146704699G > A altered the binding sites of some transcription factors (Table 4). The allele A of g.146702957G > A created a TFBS for SMARCA3 (SWI/SNF related, matrix associated, actin dependent regulator of chromatin, subfamily a, member 3) and REX1 (REX1 transcription factor; zinc finger protein 42), respectively, and the allele G created a TFBS for VMYB (v-Myb, variant of AMV v-myb). The alleles A and G of g.146704373A > G created a TFBS for BRACH (Brachyury) and NKX26 (NK2 homeobox 6, Csx2), respectively. The allele G of g.146704618A > G created two TFBSs for ZBED4 (Zinc finger, BED-type containing 4; GC-box binding sites) and SP1 (Stimulating protein 1, ubiquitous zinc finger transcription factor). The allele G of g.146704699G > A created two TFBSs for USF1 (Upstream stimulating factor 1) and ARNT (AhR nuclear translocator homodimers), and the allele A created a TFBS for FOXA1 (Forkhead box protein A1, hepatocyte nuclear factor 3-alpha (HNF-3-alpha)).

**Table 4:** Changes of transcription factor binding site (TFBS) caused by the SNP in the 5′untranslated (UTR) and flanking regions of *AGPAT3*

SNP	Sequence	Ttranscription factor	Name
g.146702957G > A	TCCCTGTGCC**G**TTTCCACTGA	VMYB	v-Myb, variant of AMV v-myb
	TCCCTGTGCC**A**TTTCCACTGA	SMARCA3	SWI/SNF related, matrix associated, actin dependent regulator of chromatin, subfamily a, member 3
		REX1	REX1 transcription factor; zinc finger protein 42
g.146704373A > G	CACGGGAAGTA**A**GGTGTGCAGT	BRACH	Brachyury
	CACGGGAAGTG**G**GTGTGCAGT	NKX26	NK2 homeobox 6, Csx2
g.146704618A > G	CTCTTCCACC**A**CCCTGGACAG		
	CTCTTCCACC**G**CCCTGGACAG	ZBED4	Zinc finger, BED-type containing 4; GC-box binding sites
		SP1	Stimulating protein 1, ubiquitous zinc finger transcription factor
g.146704699G > A	AATGGGAAAC**G**TGACAGGATT	USF1	Upstream stimulating factor 1
		ARNT	AhR nuclear translocator homodimers
	AATGGGAAAC**A**TGACAGGATT	FOXA1	Forkhead box protein A1, hepatocyte nuclear factor 3-alpha (HNF-3-alpha)

Note: The SNPs in sequences are highlighted in bold

### Exploring for luciferase activity altered by the SNPs in 5′ regulatory region

To validate the TFBS prediction results, the luciferase assay was further performed for the four SNPs (g.146702957G > A, g.146704373A > G, g.146704618A > G and g.146704699G > A) (Fig. 1b). We observed that the luciferase activities of six constructs containing g.146704373A > G, g.146704618A > G, and g.146704699G > A, were significantly higher than that of the pGL4.14 empty vector (*P* < 0.0007) and blank control (*P* < 0.0008), while g.146702957G > A did not (*P* > 0.05). Further, the luciferase activity of alleles A of g.146704373A > G and g.146704618A > G were significantly higher than that of their alleles G (*P* = 0.0004; Fig. 1b). The luciferase activity of allele G of g.146704699G > A was higher than that of allele A, while not significant (*P* > 0.05). These results indicated that the transcriptional activity of the *AGPAT3* gene significantly altered by g.146704373A > G and g.146704618A > G might be the reasons strongly impacted on FAs.

## Discussion

This study was a follow-up investigation for our previous GWAS on milk FAs in Chinese Holstein [12]. *AGPAT3* is involved in pathways related to lipid metabolism (ko00561, ko00564 and ko04072). In human, docosapentaenoic acid as the substrate of *AGPAT3* protein transfers a fatty acid in *sn*-2 position of lysophosphatic acid, a step in the phospholipid biosynthesis pathway [19]. Here, we detected that the *AGPAT3* gene mainly impacted the medium-chain milk FAs in dairy cattle.

Mammalian AGPAT catalyzed the acylation of lysophosphatidic acid to form the phosphatidic acid, which was the precursor of all glycerplipids [14]. For the AGPAT families, *AGPAT1* had significant association with milk FA CLA [20], and *AGPAT6* was strongly associated with C14:0, C16:0, C10:1, C12:1, C14:1 and C16:1 [21]. In our previous GWA studies [12, 13], *AGPAT3* gene was identified as a candidate for two milk FAs, C18index and C18:0. In this study, using an independent Chinese Holstein population that was different from the previous GWA studies, we also observed that *AGPAT3* showed a significant genetic effect on C18index and C18:0. In addition, our results revealed that the *AGPAT3* had strong associations with C6:0, C8:0 and C10:0. Overall, the previous GWASs and this study suggested that *AGPAT3* gene had significantly genetic effects on milk FAs.

Sequences-specific binding of transcription factors to the regulatory regions on the DNA is a key regulatory mechanism that affects gene expression and hence heritable phenotypic variation [22, 23]. Eukaryotic regulatory sequences, including enhancers and promoters, are typically between a hundred and several thousand base pairs in length, and can harbor many TFBSs [24]. It is essential to understand the evolution dynamics of transcription factor binding for understanding the evolution of gene regulation [25]. In this study, by prediction, g.146704373A > G changed the bindings of transcription factors (TFs) BRACH and NKX26, and g.146704618A > G altered the bindings of TFs ZBED4 and SP1. Further, we used the luciferase assay to verify that the alleles A of g.146704373A > G and g.146704618A > G strongly increased the transcription activity of the *AGPAT3* gene than the alleles G. Previous studies showed that BRACH as a regulatory factor directly activated downstream mesoderm-specific genes to exert its mesoderm-inducing effects [26], and NKX26 restrained the transcription activity of *Cx40* through the F151L missense mutation to impact the heart development [27]. ZBED4 could act as a co-repressor of nuclear hormone receptors (NHRs) by its LXXLL motifys in cones [28]. Through interfering with the recruitment of SP1 to *ZNF132* promoter region, methylation of SP1-binding site can inhibit *ZNF132* transcriptional expression to impact the tumor in the development of esophageal squamous cell carcinoma [29]. These reports have indicated that the TFs BRACH, NKX26, ZBED4 and SP1 could activate or repress the expression of their target genes. Based on our association analysis, the cows with the AA genotypes of g.146704373A > G and g.146704618A > G of *AGPAT3*, yielded significantly lower contents of C6:0 and C8:0 than those with GG genotypes. According to above, we deduced that the BRACH as a TF might activate *AGPAT3* gene transcription activity by binding to the allele A of g.146704373A > G thereby reducing the contents of C6:0 and C8:0, while, the transcription factors NKX26, ZBED4 and SP1 might have the contrary effects.

Nowadays, genomic selection is the main implication for dairy cattle breeding, where the genomic chips are used. Among the SNP markers in these chips, most of them were collected from the current SNP database and almost evenly distributed across the whole genome. Hence, g.146704373A > G and g.146704618A > G of *AGPAT3* as the potentially causal mutations could be put into the SNP chip instead of used in marker selection to increase selection efficiency in some specific dairy cattle populations to improve the contents of milk FAs.

## Conclusion

In conclusion, through a post-GWAS approach, our study firstly indicated there were significant genetic associations between the *AGPAT3* gene and milk FAs in dairy cattle. Further, we found that two SNPs in 5′ regulatory region (g.146704373A > G and g.146704618A > G) changed the transcriptional activity of *AGPAT3* implying their potential causal function. These findings provided important molecular information for dairy cattle breeding.

## Supplementary Information

**Additional file 1: Table S1.** PCR primers information of *AGPAT3* gene

**Additional file 2: Table S2.** Additive (a), dominant (d) and allele substitution (α) effects of 17 SNPs on milk fatty acid traits of *AGPAT3* gene in Chinese Holstein cows

## Data Availability

All relevant data are available within the article and its supplementary information.

## Abbreviations

a: Additive
AGPAT: 1-Acylglycerol-sn-glycero 3-phosphate acyltransferase
AGPAT3: 1-Acylglycerol-3-phosphate O-acyltransferase 3
ARNT: AhR nuclear translocator homodimers
BRACH: Brachyury
d: Dominant
FA: Fatty acid
FOXA1: Forkhead box protein A1, hepatocyte nuclear factor 3-alpha (HNF-3-alpha)
GWAS: Genome-wide association study
HEK: Human embryonic kidney
LD: Linkage disequilibrium
NKX26: NK2 homeobox 6, Csx2
PCR: Polymerase chain reaction
REX1: REX1 transcription factor; zinc finger protein 42
SFA: Saturated fatty acids
SMARCA3: SWI/SNF related, matrix associated, actin dependent regulator of chromatin, subfamily a, member 3
SNP: Single nucleotide polymorphism
SP1: Stimulating protein 1, ubiquitous zinc finger transcription factor
TFBS: Transcription factor binding site
UFA: Unsaturated fatty acid
USF1: Upstream stimulating factor 1
UTR: Untranslated region
VMYB: v-Myb, variant of AMV v-myb
ZBED4: Zinc finger, BED-type containing 4, GC-box binding sites
α: Substitution
